# The Influence of Interleukin *17A* and *IL17F* Polymorphisms on Chronic Periodontitis Disease in Brazilian Patients

**DOI:** 10.1155/2015/147056

**Published:** 2015-08-03

**Authors:** Joana Maira Valentini Zacarias, Emília Ângela Sippert, Patrícia Yumeko Tsuneto, Jeane Eliete Laguila Visentainer, Cléverson de Oliveira e Silva, Ana Maria Sell

**Affiliations:** ^1^Post Graduation Program of Biosciences and Physiopathology, Biomedicine and Clinical Analysis Department, Maringá State University, 87020-900 Maringá, PR, Brazil; ^2^Biomedicine and Clinical Analysis Department, Maringá State University, 87020-900 Maringá, PR, Brazil; ^3^Basic Health Sciences Department, Maringá State University, 87020-900 Maringá, PR, Brazil; ^4^Dentistry Department, Maringá State University, 87020-900 Maringá, PR, Brazil

## Abstract

A case-control study was conducted on patients with chronic periodontitis (CP) and healthy controls with the aim of evaluating possible association between interleukin *17A (IL17A)* G197A (rs2275913) and *IL17F* T7488C (rs763780) polymorphisms and periodontitis. Genotypes were determined by PCR-RFLP method. Statistical analyses were conducted using the OpenEpi and SNPStas software to calculate Chi-square with Yates correction or Fisher's exact tests, odds ratios (OR), and 95% confidence intervals (CIs). SNPStas software was used to calculate Hardy-Weinberg equilibrium. *IL17A* AA genotype was more frequent in patients with chronic periodontitis (CP) in the codominant and recessive models (*P* = 0.09; OR = 2.53 and *P* = 0.03; OR = 2.46, resp.), the females with CP (*P* = 0.01, OR = 4.34), Caucasoid patients with CP (*P* = 0.01, OR = 3.45), and nonsmoking Caucasian patients with CP (*P* = 0.04, OR = 3.51). The *IL17A* A allele was also more frequent in Caucasians with CP (*P* = 0.04, OR = 1.59). *IL17F* T7488C polymorphism was not associated with chronic periodontitis. In these patients from Southern Brazil, the *IL17A* rs2275913 polymorphisms, *IL17A* AA genotype, and the A allele were associated with a susceptibility to chronic periodontitis.

## 1. Introduction

Periodontitis is a chronic inflammatory disease that affects the tooth supporting tissue and destroys alveolar bone. It is the most frequent cause of tooth loss in the adult [1]. Epidemiologic studies suggest that up to 60% of the population is affected by the common form of the disease, termed chronic periodontitis (CP) [1, 2]. Periodontitis has been said to have interaction with a number of common human diseases like diabetes mellitus or rheumatoid arthritis. Epidemiological data has confirmed that diabetes has been a major risk factor for the onset of periodontitis and showed a clear relationship between degree of hyperglycemia and severity of periodontitis [3]. Other studies show that patients with rheumatoid arthritis had a higher incidence of periodontitis compared to healthy controls [4, 5], though the mechanism of periodontitis that interacted with other diseases was not very clear.

Periodontitis is a multifactorial disease and as such, the significant elements include not only the presence of pathogenic bacteria and the immune mechanism, but also the genetic predisposition [6]. From a pathophysiology perspective, periodontitis is the result of host-mediated inflammatory damage of the supporting tissues triggered in response to the microbial infection [7, 8]. More than 700 different bacterial species have been shown to inhabit periodontal biofilms [9] and some species are currently considered to be causally associated with periodontitis; these include Gram-negative species, such as anaerobic* Porphyromonas gingivalis*, a principal pathogen in chronic periodontitis [10].

Specific cytokines are important in the pathogenic process of the periodontitis [8, 11–13]. Interleukin-17 (IL-17) is a proinflammatory cytokine secreted by activated T cells [14]. The IL-17 family contains six members, IL-17A, IL-17B, IL-17C, IL-17D, IL-17E (or IL-25), and IL-17F, and five receptors, IL-17RA–RD and SEF. Interleukin-17A is most homologous to IL-17F and the genes encoding them are proximally located on chromosome 6p12 [15]. The IL-17F activity is similar to IL-17A but significantly weaker and is related to inducing the expression of various cytokines, chemokines, matrix metalloproteinases, antimicrobial peptides, and adhesion molecules by human fibroblasts, airway epithelial cells, and vein endothelial cells [16].

Th17 cells were a distinct T lineage that do not share developmental pathways with either Th1 or Th2 cells [17]. Th17 cells have been linked to several autoimmune disorders and are also linked to the development of pathological inflammatory disorders. However Th17 cells are physiologically found in the lamina propria of the intestine [17, 18]. Cytokines related with Th17, such as IL-17 and IL-22, are crucial for host protection against many extracellular pathogens. IL-17 stimulates the production and expression of TNF-alpha and IL-1 beta by human macrophages [19] and induces production of IL-1 beta in osteoblasts [20]. Thus, IL-17 was found to contribute to inflammatory bone pathology as in rheumatoid arthritis and inflammatory bowel diseases and was centrally involved in numerous autoimmune disorders [21–25].

IL-17 cytokine can stimulate fibroblasts, epithelial and endothelial cells, to produce IL-6, CXCL8/IL-8, and prostaglandin E2 (PGE2) [26]. IL-17 also induces the expression of receptor activator of nuclear factor kappa B ligand (RANKL) on osteoblasts and stimulates the differentiation and activation of osteoclasts, which can influence bone resorption mediated by these cells [22]. Many studies have demonstrated the presence of IL-17 in periodontal tissues, crevicular gingival fluid, saliva, and plasma of patients with periodontal disease [27–30].

In order to investigate whether* IL17A* and the* IL17F* polymorphisms are associated with chronic periodontal disease and understand its immunopathogenesis, this study aimed at evaluating the* IL17A* G197A (rs2275913) and* IL17F* T7488C (His161Arg, rs763780) polymorphisms in patients with chronic periodontitis and in a healthy group who had undergone dental care in the North/Northwest of the state of Paraná, Southern Brazil.

## 2. Materials and Methods

### 2.1. Sample Selection

A total of 313 individuals were selected from those who sought dental treatment in the dental clinics of the Maringa State University (UEM) and Inga University (UNINGÁ) from January 2012 to December 2014. After taking patient's medical records, clinical periodontal examinations were conducted by two examiners. Clinical parameters of probing depth (PD) and clinical attachment level (CAL) were examined at six sites (mesiovestibular, vestibular, distovestibular, mesiolingual, lingual, and distolingual) of each tooth, as was bleeding on probing (BOP).

After the periodontal examination, participants were assigned to two different groups: the chronic periodontitis group (*N* = 140) composed of individuals who had at least 5 sites in different teeth with PD ≥5 mm, CAL ≥3 mm, and more than 25% of BOP and the control group (*N* = 173), formed by individuals who did not have sites with reduced CAL, displayed a PD of less than 4 mm, and exhibited less than 25% of BOP. Both patients and controls were from the North and Northwest regions of the state of Paraná (between 22°29′30′′–26°42′59′′S and 48°02′24′′–54°37′38′′W), Southern Brazil, over 30 years of age, from all ethnic groups, and with at least 20 teeth in the oral cavity. Information on the patient's ethnic background and smoking history was obtained by interviewing the individual (anamnesis). Exclusion criterions were patients and control subjects with diabetes mellitus and acute infections and patients with aggressive periodontitis and who had periodontal treatment in the last 6 months.

All individuals who agreed to participate in this research were informed about the nature of the study and signed an informed consent form. This study was approved by the Human Research Ethics Committee of the Maringa State University (UEM: number 719/2011, 02/12/2011).

### 2.2. DNA Extraction

To extract the DNA, the buffy coat was obtained from 4 mL of peripheral blood collected in EDTA by centrifugation (210 g for 15 min). The DNA was extracted using the salting-out method [31]. The concentration and quality of the DNA were analyzed by optical density in a Thermo Scientific Nanodrop 2000 apparatus (Wilmington, USA).

### 2.3. Genotyping Analysis

Single nucleotide polymorphisms (SNP) to* IL17A* G197A (rs2275913) and* IL17F* T7488C (rs763780, His161Arg) genotyping were performed by Polymerase Chain Reaction-Restriction Fragment Length Polymorphism (PCR-RFLP) [32] with some modifications. Primer sequences for* IL17A* G197A were sense 5′-AACAAGTAAGAATGAAAAGAGGACATGGT-3′ and antisense 5′-CCCCCAATGAGGTCATAGAAGAATC-3′ and for* IL17F* T7488C were sense 5′-ACCAAGGCTGCTCTGTTTCT-3′ and antisense 5′-GGTAAGGAGTGGCATTTCTA-3′. PCR amplification was performed in a total volume of 30 *μ*L mixture containing 100 ng genomic DNA, 1.0 *μ*M of each primer, 200 *μ*M of each dNTP, 2.0 mM of MgCl_2_, 3 *μ*L 10x PCR buffer, and 1.5 U Taq DNA polymerase (Invitrogen Life Technologies, Grand Island, NY, USA). PCR products were digested for one hour at 37°C with* XagI* (Fermentas, Canada) for* IL17A* G197A and* NlaIII* (New England Biolabs) for* IL17F* T7488C and then separated by electrophoresis on 3% agarose with SYBR Green (Invitrogen Life Technologies, Grand Island, NY, USA).

### 2.4. Statistical Analysis

Allele and genotype frequencies of* IL17A* G197A and* IL17F* T7488C were obtained by direct counts. The association between genetic polymorphisms and chronic periodontitis was evaluated using the Chi-square test with Yates correction or the Fisher's exact test and the correlation was deemed present by an odds ratio with 95% confidence intervals only for significant *P* values. Adjusting the genotypic differences for the effect of age, gender, and smoking status was applied. All tests were carried out using a significance level of 5%. For these analyses and calculating Hardy-Weinberg equilibrium OpenEpi program Version 2.3.1 and SNPStas software (http://bioinfo.iconcologia.net/index.php) were used.

## 3. Results

Polymorphisms in the* IL17A* G197A (rs2275913) and* IL17F* T7488C (rs763780) were analyzed in 140 CP patients and 173 control subjects. Most participants were female (61.0%), Caucasian (64.5%), and nonsmokers (58.5%). The characteristics of patient and control subjects are described in Table 1.

Differences were noted in smoking history when the groups were compared, and to eliminate the smoking as a confounding factor, all analyses were also done in the nonsmoker patients versus nonsmoker controls. There was no significant difference with respect to gender, age, and ethnic background distributions.

Genotype distribution of* IL17A* G197A and* IL17F* T7488C in CP patient and control groups was consistent with the Hardy-Weinberg equilibrium (*P* > 0.05). The* IL17A* and* IL17F* were not in linkage disequilibrium (*P* = 0.51).

The genotype and allele frequencies distributions are summarized in Table 2. There were significant differences in the codominant and recessive models for* IL17A* AA genotype between all CP patients and controls. No significant difference was observed for* IL17F* in the recessive, dominant, and codominant models.

We analyzed* IL17A* and* IL17F* genotype and allele frequencies in CP and controls after stratifying according to gender and ethnic background. No significant difference was observed for* IL17F* polymorphism after stratification. However* IL17A* AA genotype was more frequent in female CP patients (16.2%* versus* 4.3%, *P* = 0.01, OR = 4.34, and 95% CI: 1.46–12.87) (Table 3).* IL17A* AA genotype and A allele were more frequent in Caucasoid CP patients than in controls (17.8%* versus* 5.9%, *P* = 0.01, OR = 3.45, and 95% CI: 1.34–8.88, and 38.7%* versus* 28.4%, *P* = 0.04, OR = 1.59, and 95% CI: 1.05–2.42, resp.). Difference was also found for* IL17A* AA genotype that was more frequent in the Caucasian and nonsmoking CP patients than in the Caucasian nonsmoking controls (22.2%* versus* 8.1%, *P* = 0.048, OR = 3.51, and 95% CI: 1.17–10.55) (Table 4).

## 4. Discussion

In this study we investigated a possible role of* IL17A* and* IL17F* polymorphisms in immunopathogenic mechanism for CP in a Southern Brazilian population. We observed that the* IL17A* 197AA genotype was more frequent in patients with chronic periodontitis (CP), females with CP, and the Caucasian and nonsmoking Caucasian patients with CP than in respective controls, and this could be correlated to the risk of disease. The* IL17F* T7488C polymorphism was not associated with CP risk in this population.

In a case-control study, pairing between study subjects is necessary in order to avoid bias in the final results. CP can be related to individual risk factors such as stress, diabetes, osteoporosis, and arthritis [3–5, 33–37] and, in this studied population, they were considered confounding factors and were an exclusion criterion. On the other hand, smoking habits, also a predisposition factor with oral diseases and especially in the chronic periodontitis [38–41], were more frequent in CP patients (smoker + ex-smoker: 60%* versus* 40%; *P* < 0.001, OR = 4.12, and 95% CI: 2.56–6.69); thus, to exclude smoking as a predisposing factor, statistical analyses were performed in all the individuals as well as in the nonsmokers CP versus nonsmokers in control group, and stratifying by smoking habits was not considered. Some studies alert that gender can be a confounding factor to periodontitis [42] although others showed the higher frequency in male was principally related to personal hygiene habits [43]; furthermore, it was revealed that there was no meaningful relation to the role of IL-17 polymorphism in men and in women when compared to each other [44]. With regard to ethnic background, the distributions of patients and controls were similar: this is important because* IL17A* and* IL17F* alleles and genotypes differ in different populations (http://www.snpedia.com/index.php/Rs2275913 and /Rs763780) and Brazilians are an admixed population. Although ethnicity was not a confounding variable, the analyses were done after stratification by the Caucasian, Black, and mixed population.

In the present report* IL17F* was not associated with periodontal disease in all the patients nor in nonsmoking patients and controls who were investigated in the recessive, dominant, and codominant models. However,* IL17A* 197AA genotype was more frequent in CP patients, in female patients, and in the Caucasian CP patients as well as in the nonsmoking Caucasian patients with CP. Furthermore,* IL17A* 197A allele was also more frequent in Caucasians with CP. These results suggest that* IL17A* 197AA genotype and A allele could be related to higher risk for the development of chronic periodontitis. In our Caucasian group with CP the* IL17A* 197G allele was less frequent and could be a resistant factor. The polymorphism in the promoter region of cytokines may be related to higher expression of the specific cytokine. According to Espinoza et al. [45] the* IL17A* 197A allele correlates to more efficient IL-17 secretion and higher affinity for the nuclear factor of activated T cells (NFAT), which is a critical regulator of the* IL17* promoter gene [46].

The* IL17A* 197AA genotype was associated with tumorigenesis susceptibility as in gastric cancer [47, 48], breast cancer [49], and cervical cancer [50], as well as autoimmunity diseases such as ulcerative colitis [51, 52] and rheumatoid arthritis [53]. As for periodontitis diseases, only two previous studies in the Brazilian patients were reported. Corrêa et al. [54] showed similar results to our study:* IL17A* 197AA genotype and A allele were associated with worse clinical and inflammatory periodontal parameters. Saraiva et al. [55] conducted another study on chronic periodontitis in Brazil and severe periodontitis patients with the aim of investigating the phenotypic expression of* IL17A* and the polymorphisms of* IL17A* and* IL17F* within different clinical forms and severity of the disease. However, differently to our and Corrêa et al.'s results [54], their data suggested that the IL-17 and* IL17A* A allele were associated with the absence of periodontal disease, and the* IL17A* GG genotype and G allele were associated with risk factors.

According to the distribution of* IL17A* genotypes frequencies in our control group,* IL17A* AA was 7.0%,* IL17A* AG was 42.7%, and* IL17A* GG was 50.3%, similar to those in the study conducted by Saraiva et al. [55] which were 10% for AA, 44% for AG, and 46% for GG but very different from those of Corrêa et al. [54] which were 25.9% for AA, 14.81% for AG, and 59.26% for GG.

The* IL17F* T7488C polymorphism was not associated with chronic periodontitis in this study. This result was similar to Corrêa et al. [54] and Saraiva et al. [55] in CP Brazilian patients.* IL17F* has been associated with several diseases like asthma, Crohn's disease, multiple sclerosis, inflammatory bowel disease, autoimmune thyroid diseases, tuberculosis, and dilated cardiomyopathy as well as a high risk of recurrent pregnancy loss [51, 56–61]. According to the literature, the IL-17F activity is similar to IL-17A but significantly weaker, and the variant form of IL-17 protein (His121Arg) suppresses the expression and the activity of wild type [16, 62].

According to the* IL17F* genotype frequency distribution in our control group, we had 8.7% for* IL17F* TC and 91.3% for* IL17F* TT; the* IL17F* CC genotype was absent in our control population. Again, it was similar to the study conducted by Saraiva et al. [55] which found 6.4% for TC and 93.6% for TT and did not find the* IL17F* CC genotype. However, the distribution of genotype frequencies found by Corrêa et al. [54] was also very different: they found 16.66% for* IL17F* TC, 56.66% for* IL17F* TT, and 23.33% for* IL17F* CC in their Brazilian control group. The distribution of genotype frequency in our populations was consistent with a low frequency of polymorphic genotype of* IL17F* T7488C found in other populations (http://www.snpedia.com/index.php/Rs763780).

CP progression was related to a host inflammatory response that mediates tissue damage, and several previous studies had been relating immune genetic factors to CP disease, especially cytokines genes polymorphism [63–69]. The IL-17, a proinflammatory cytokine, was detected in periodontal tissues, crevicular gingival fluid, saliva, and plasma of patients with periodontal disease [27–30, 70]. IL-17, especially when combined with IFN-gamma, may have a role in immune modulation through stimulation of human gingival fibroblasts in periodontal disease [71]. This occurs by triggering the release of other proinflammatory, metalloproteinase, and neutrophil-mobilizing cytokines [47, 49, 51, 72] and having effects on osteoclasts maturity as a stimulating factor [73, 74]. IL-17A and IL-17F have a very similar amino acid sequence and both play similar functions and have the ability to induce chemokines which is crucial to the neutrophil recruitment and activation [75], the first wall in the periodontal diseases. Thus, understanding* IL17* polymorphism is necessary in order to infer the role of IL-17 in the CP immunopathogenesis.

In these Southern Brazilian patients, the* IL17A* rs2275913 polymorphisms, AA genotype, and A allele were associated with a susceptibility to chronic periodontitis disease, in females with CP, and in the Caucasian and nonsmoking Caucasian patients. Furthermore, the possible immunopathogenic mechanism would be investigated in the future through histological studies.

## 5. Conclusion

In conclusion we can infer that* IL17A* G197A rs2275913 polymorphism,* IL17A* AA genotype, and A allele could be associated with a susceptibility to chronic periodontitis but no evidence showed for risk or protection associations for* IL17F* T7488C rs763780. Additional studies are necessary for understanding the functional role of rs2275913 polymorphisms in chronic periodontitis.

## Figures and Tables

**Table 1:** Characteristics of patients with chronic periodontitis (CP) and controls.

	CP patients	Controls	*P*	OR (95% CI)
	*n* (%)	*n* (%)		

	*N* = 140	*N* = 173		

Gender				
Male	66 (47)	56 (32)		
Female	74 (53)	117 (68)		
Age				
Mean ± SD (year)	47.03 ± 9.21	45.61 ± 9.18		
Ethnic origin				
Caucasian	84 (60)	118 (68)		
Mixed	36 (26)	40 (23)		
Black	18 (13)	15 (9)		
Not declared	2 (1)	0		
Smoking				
Nonsmokers	56 (40)	127 (73)	<0.001	0.24 (0.149–0.39)
Smokers	33 (24)	20 (12)	0.008	2.78 (1.33–5.78)
Ex-smokers	51 (36)	26 (15)	<0.001	3.24 (1.89–5.56)

Nonsmoker	*N* = 56	*N* = 127		

Gender				
Male	20 (35.7)	33 (26.0)		
Female	36 (64.28)	94 (74.0)		
Age				
Mean ± SD (year)	46.5 ± 9.3	45.5 ± 9.9		
Ethnic origin				
Caucasian	36 (64.28)	86 (67.7)		
Mixed	16 (28.6)	28 (22.0)		
Black	3 (5.4)	13 (10.3)		
Not declared	1 (1.8)	0		

*n*: number; *P* = *P* value; OR: odds ratio; CI: confidence interval.

**Table 2:** Genotypes and allele distribution of *IL17A* rs2275913 and *IL17F* rs763780 in the patients with chronic periodontitis and controls from Southern Brazil.

Genotype	All subjects			Nonsmokers		
	Patients	Controls		Patients	Controls	
	*N* = 140 *n* (%)	*N* = 173 *n* (%)		*N* = 56 *n* (%)	*N* = 127 *n* (%)	
rs2275913						
GG	67 (47.9)	87 (50.3)	Ref.	25 (44.6)	61 (48.0)	Ref.
AA	18 (12.9)	12 (7.0)	*∗*	9 (16.1)	10 (7.9)	
AG	55 (39.2)	74 (42.7)		22 (39.3)	56 (44.1)	
AA/AG	73 (52.1)	86 (49.7)		31 (55.4)	66 (52.0)	
GG/AG	122 (87.1)	99 (57.2)		47 (83.9)	117 (92.1)	
Allele						
A	91 (32.5)	98 (28.3)		40 (35.7)	76 (30.0)	
G	189 (67.5)	248 (71.7)		72 (64.3)	178 (70.0)	
rs763780						
TT	125 (89.3)	158 (91.3)	Ref.	49 (87.5)	116 (91.3)	Ref.
CC	1 (0.7)	0		1 (1.8)	0	
TC	14 (10.0)	15 (8.7)		6 (10.7)	11 (8.7)	
CC/TC	15 (10.7)	15 (8.7)		7 (92.9)	11 (8.7)	
TT/TC	139 (99.3)	173 (100)		55 (98.2)	127 (100)	
Allele						
T	264 (94.3)	331 (95.7)		104 (92.9)	243 (95.7)	
C	16 (5.7)	15 (4.3)		8 (7.1)	11 (4.3)	

^*∗*^Codominant model: *P* = 0.09; OR = 2.53; 95% CI = 1.07–5.99; recessive model: *P* = 0.03; OR = 2.46; 95% CI = 1.08–5.59.

**Table 3:** *IL17A* genotype and allele frequencies in chronic periodontitis Brazilian patients and controls stratified according to gender^*∗*^ in the total samples and nonsmoking individuals.

	*IL17A* rs2275913	CP patients *n* (%)	Controls *n* (%)
Female	Genotype	*N* = 74	*N* = 117
	GG	36 (48.7)	63 (53.8)
	AA^*∗∗*^	12 (16.2)	5 (4.3)
	GA	26 (35.1)	49 (41.9)
	Allele		
	A	50 (33.8)	59 (25.2)
	G	98 (66.2)	175 (74.8)

Nonsmoker female	Genotype	*N* = 14	*N* = 33
	GG	6 (42.9)	11 (33.3)
	AA	2 (14.2)	5 (15.2)
	GA	6 (42.9)	17 (51.5)
	Allele		
	A	10 (35.7)	27 (40.9)
	G	18 (64.3)	39 (59.1)

CP: chronic periodontitis; ^*∗*^only significant differences were showed; ^*∗∗*^ *P* = 0.01, OR = 4.34, and 95% CI: 1.46–12.87.

**Table 4:** *IL17A* genotype and allele frequencies in chronic periodontitis Brazilian patients and controls stratified according to ethnic group^*∗*^.

	*IL17A* rs2275913	CP patients *n* (%)	Controls *n* (%)	*P*	OR (95% CI)
Caucasian	Genotype	*N* = 84	*N* = 118		
	GG	34 (40.5)	58 (49.2)		
	AA	15 (17.8)	7 (5.9)	0.01	3.45 (1.34–8.88)
	GA	35 (41.7)	53 (44.9)		
	Allele				
	A	65 (38.7)	67 (28.4)	0.04	1.59 (1.05–2.42)
	G	103 (61.3)	169 (71.6)	0.04	0.63 (0.41–0.96)

Nonsmoker Caucasian	Genotype	*N* = 36	*N* = 86		
	GG	15 (41.7)	41 (47.7)		
	AA	8 (22.2)	7 (8.1)	0.048	3.51 (1.17–10.55)
	GA	13 (36.1)	38 (44.2)		
	Allele				
	A	29 (40.3)	52 (30.2)		
	G	43 (59.7)	120 (69.8)		

CP: chronic periodontitis; ^*∗*^only significant differences were showed.
